# Lebertransplantation in der Schweiz 2020

**DOI:** 10.1007/s43472-020-00025-9

**Published:** 2020-11-11

**Authors:** Katharina Staufer, Antonio Galante, Andrea De Gottardi

**Affiliations:** 1 Universitätsklinik für Viszerale Chirurgie und Medizin, Inselspital, Universitätsspital Bern, Freiburgstrasse, 3010 Bern, Schweiz; 2 Servizio di Gastroenterologia e Epatologia, Ente Ospedaliero Cantonale, Università della Svizzera Italiana, Lugano, Schweiz

**Keywords:** Leberzirrhose, Akutes Leberversagen, Immunsuppression, Hepatozelluläres Karzinom, Nachsorge, Cirrhose du foie, Insuffisance hépatique aiguë, Immunosuppression, Carcinome hépatocellulaire, Suivi médical, Cirrosi epatica, Insufficienza epatica acuta, Immunosoppressione, Carcinoma epatocellulare, Post-terapia

## Abstract

Die Lebertransplantation ist in den letzten beinahe 40 Jahren zu einer etablierten Therapie der fortgeschrittenen Leberzirrhose, des akuten Leberversagens sowie gewisser auf die Leber beschränkter Tumorerkrankungen geworden und stellt somit für viele unserer Patientinnen und Patienten eine lebensrettende Behandlungsmöglichkeit dar. Leider jedoch ist der Zugang zu einer Lebertransplantation dadurch limitiert, dass nicht für alle Patientinnen und Patienten ausreichend Spenderorgane zur Verfügung stehen. Der folgende Artikel fasst die wichtigsten Punkte zur Indikation, Abklärung vor Transplantation sowie zum Management nach der Transplantation zusammen.

## Résumé

Depuis presque 40 ans à présent, la greffe du foie est devenue un traitement établi de la cirrhose avancée du foie, de l’insuffisance hépatique aiguë et de certaines maladies tumorales limitées au foie, et elle est donc un traitement qui sauve la vie à un grand nombre de nos patients. Un aspect cependant regrettable est que l’accès à une greffe du foie reste limité par le fait que les dons d’organes ne sont pas assez nombreux pour tous les patients. L’article suivant résume les points les plus importants concernant l’indication, les investigations avant la transplantation et la prise en charge après la transplantation.

## Riassunto

Il trapianto di fegato è diventato una terapia consolidata per la cirrosi epatica avanzata, l’insufficienza epatica acuta e alcuni tumori circoscritti al fegato ormai da quasi 40 anni, e rappresenta quindi un’opzione terapeutica salvavita per molti dei nostri pazienti. Purtroppo, però, l’accesso al trapianto di fegato è limitato dal fatto che gli organi disponibili per il trapianto non sono sufficienti per tutti i pazienti. Il seguente articolo riassume i punti più importanti riguardanti le indicazioni, le spiegazioni della fase pre-trapianto e la gestione del post-trapianto.

## Einleitung

Die erste erfolgreiche Lebertransplantation weltweit wurde 1967 durch Thomas Starzl in den USA durchgeführt. Damit die Lebertransplantation zu einem etablierten Therapieverfahren werden konnte, waren jedoch, neben der Optimierung des chirurgischen Eingriffs selbst, zahlreiche weitere Entwicklungen erforderlich, allen voran die Entdeckung und spätere Markteinführung von Cyclosporin A. Die dadurch deutlich verbesserte Immunsuppression machte erstmals Langzeitüberleben nach Lebertransplantation möglich. Das 10-Jahres-Überleben nach Lebertransplantation beträgt heutzutage etwa zwischen 50 und 80 % in Abhängigkeit von der Indikation zur Transplantation (www.eltr.org. Zugegriffen: September 2020).

Die erste Lebertransplantation in der Schweiz wurde am Inselspital in Bern 1983 durchgeführt. Mittlerweile haben sich 3 Zentren für Lebertransplantation in der Schweiz etabliert, Genf, Zürich und Bern. Die Organallokation erfolgt nach dem Transplantationsgesetz nach objektiven Kriterien über Swisstransplant, die Schweizer Nationale Stiftung für Organspende und Transplantation. Swisstransplant ist zudem zuständig für die Führung der nationalen Warteliste.

## Indikationen zur Lebertransplantation

Die Indikation für eine Lebertransplantation besteht bei einer zunehmenden Einschränkung der Leberfunktion unabhängig von der zugrunde liegenden akuten und/oder chronischen Erkrankung. Das Prinzip einer Aufnahme auf die Warteliste zur Lebertransplantation ist, dass die Überlebenswahrscheinlichkeit und/oder die Lebensqualität der Patientinnen und Patienten mit Transplantation größer ist als ohne Transplantation. Aufgrund des Organmangels kann jedoch nur ein Teil aller Patientinnen und Patienten die lebensrettende Transplantation erhalten. In der Schweiz wurden im Jahr 2019 168 Lebertransplantationen durchgeführt. Die Zahl der Transplantationen stieg somit weiter an (2015: 136, 2016: 108, 2017: 143, 2018: 156), jedoch stieg auch die Anzahl der Patientinnen und Patienten auf der Warteliste (427 Patientinnen und Patienten auf der Warteliste im Jahr 2019; https://www.swisstransplant.org/de/swisstransplant/publikationen/jahresberichte/. Zugegriffen: September 2020).

Die Hepatitis-C-assoziierte Leberzirrhose stellte lange Zeit die Hauptindikation zur Lebertransplantation dar. Durch die Markteinführung hochwirksamer Substanzen („direct acting antiviral agents“, DAA) ab 2013, die zumeist eine Heilung der Hepatitis C erreichen können, kam es in den letzten Jahren zu einer Verschiebung der Transplantationsindikationen. Insbesondere die mit einer Nicht-alkoholischen Fettlebererkrankung (NAFLD) assoziierte Leberzirrhose, aber auch die Alkohol-assoziierte Leberzirrhose sowie das hepatozelluläre Karzinom (HCC) stehen heutzutage im Vordergrund. Die wichtigsten Indikationen zur Lebertransplantation sind in Tab. 1 dargestellt.

**Table 1:** 

**Chronische Lebererkrankungen/Leberzirrhose**
*Parenchymatöse Lebererkrankungen*
Chronische Hepatitis B (CHB), Hepatitis B/D-Koinfektion Chronische Hepatitis C (CHC) Alkoholassoziierte Leberzirrhose (ALD) Nichtalkoholische Fettlebererkrankung (NAFLD)
*Autoimmune und cholestatische Lebererkrankungen*
Autoimmune Hepatitis (AIH) Primär biliäre Cholangitis (PBC) Primär (und sekundär) sklerosierende Cholangitis (PSC/SSC)
*Neoplasien*
Hepatozelluläres Karzinom (HCC; inklusive fibrolamellares HCC) Cholangiokarzinom (CCA; *z.* *T. Gegenstand laufender Studien)* Hämangioendotheliom (EHE) Isolierte Lebermetastasen bei neuroendokrinem Tumor (NET) Isolierte Lebermetastasen bei kolorektalem Karzinom (experimentelle Indikation) Adenomatose der Leber
*Hereditäre Lebererkrankungen*
α1-Antitrypsin-Mangel Hämochromatose Morbus Wilson
*Vaskuläre Lebererkrankungen*
Budd-Chiari-Syndrom
*Komplikationen der Leberzirrhose*
Hepatopulmonales Syndrom
**Seltene Indikationen**
Zystische Fibrose-assoziierte Lebererkrankung Primäre Hyperoxalurie Typ 1 Transthyretinassoziierte familiäre Amyloidpolyneuropathie (hATTR) Morbus Gaucher Glykogenose Typ 1 Adulte polyzystische Degeneration der Leber (APDL) Biliäre Atresie Caroli-Syndrom Crigler-Najjar-Syndrom Tyrosinämie Hepatoblastom Amyloidose
**(Sub)akutes Leberversagen**
Medikamente (z. B. Paracetamolintoxikation, Antibiotika etc.) Toxine (Pilze, z. B. Amanita) Morbus Wilson Autoimmunhepatitis Virushepatitis Akutes Budd-Chiari-Syndrom

Um die Dringlichkeit einer Transplantation einzuschätzen, wird der Model-of-endstage-liver-disease(MELD)-Score als Standard verwendet. Der MELD-Score beschreibt die Wahrscheinlichkeit, im Endstadium einer Lebererkrankung innerhalb von 3 Monaten zu versterben [1]. Das Hauptziel der MELD-basierten Organallokation ist die Senkung der Mortalität auf der Warteliste. Für eine begrenzte Anzahl der Patienten reflektiert jedoch der MELD-Score die Dringlichkeit der Transplantation nicht adäquat. Dazu gehören beispielsweise Patientinnen und Patienten mit bestimmten cholestatischen Lebererkrankungen, hepatopulmonalem Syndrom oder Neoplasien. Aus diesem Grund können in bestimmten Fällen Punkte für eine sog. „*standard exception*“ (SE) oder „*non standard exception*“ (NSE) zusätzlich zugeteilt werden. Ausgenommen von der MELD-Allokation sind hochdringliche Lebertransplantationen. Bei Vorliegen eines akuten Leberversagens finden andere Kriterien Anwendung, darunter die King’s-College-Kriterien [2] und die Clichy-Kriterien [3].

## Kontraindikationen zur Lebertransplantation

Vor Aufnahme auf die Warteliste für eine Lebertransplantation ist es erforderlich, nicht nur die Indikation korrekt zu prüfen, sondern auch Kontraindikationen auszuschliessen. Diese können durch die Lebererkrankung selbst oder durch schwere Komorbiditäten bedingt sein. Die medizinischen Zustände, die das Operationsrisiko erheblich erhöhen oder den längerfristigen Erfolg der Transplantation infrage stellen, sind als Kontraindikationen definiert. Tab. 2 zeigt einen Überblick über die wichtigsten Kontraindikationen. Auch eine fraglich unzureichende oder sogar fehlende Zusammenarbeit der Patientin oder des Patienten mit dem behandelnden Ärzteteam (Compliance, Adhärenz) kann als eine Kontraindikation betrachtet werden. Tritt bei einer Patientin oder einem Patienten auf der Warteliste eine vorübergehende Kontraindikation auf, wird sie oder er temporär als nichttransplantierbar eingestuft und bei der Organvermittlung vorläufig nicht berücksichtigt. Beispiele dafür sind floride, nicht kontrollierte Infektionen. Besteht die Kontraindikation nicht mehr, wird der/die Patient/in umgehend wieder in die aktive Warteliste mit der dann aktuell gegebenen Dringlichkeit als transplantierbar aufgenommen.

**Table 2:** 

Alter 70 Jahren *(relative Kontraindikation, entscheidend dabei ist das biologische Alter!)*
Body-Mass-Index (BMI) 40 *(relative Kontraindikation)*
Florider Alkohol- oder Drogenabusus *(Opiatsubstitution ist keine generelle Kontraindikation)*
Nichtkurativ behandelte extrahepatische Tumorerkrankung
HCC mit Makrogefässinvasion oder Fernmetastasierung
Fortgeschrittenes CCA mit Makrogefässinvasion oder Fernmetastasierung
Hämangiosarkom
Akute unkontrollierte Infektionen *(z.* *B. Sepsis, AIDS)*
Schwerwiegende Erkrankungen anderer Organe *(inklusive schwere kardiale oder pulmonale Funktionseinschränkung, schwere pulmonalarterielle Hypertonie)*
Vorhersehbare schwerwiegende operativ-technische Probleme *(z. B. langstreckige Pfortaderthrombose inklusive Konfluens, relative Kontraindikation)*
Fehlende Compliance/Adhärenz des Patienten
Schwere, nichtbehandelte psychische Erkrankungen

## Abklärung zur Lebertransplantation

Vor einer Aufnahme auf die Warteliste ist somit eine umfassende medizinische sowie psychologisch-psychiatrische Abklärung erforderlich. Diese kann im Rahmen einer Kurzhospitalisation oder aber auch in seltenen Fällen ambulant erfolgen. Die Tab. 3 fasst die minimal erforderlichen Untersuchungen zusammen.

**Table 3:** 

Kardiopulmonale Abklärung	Spirometrie inklusive Diffusionskapazität Transthorakale Echokardiographie inklusive schätzungsweise Bestimmung des pulmonalarteriellen Drucks Ggf. kardialer Belastungstest/Koronarangiographie
Ausschluss extrahepatischer Malignome (stratifiziert nach Alter und individuellem Risiko)	Gastroskopie Koloskopie Gynäkologische Abklärung, ggf. inklusive Mammographie Urologische Abklärung bei Männern Dermatologische Abklärung Röntgenuntersuchung des Thorax und Sonographie des Abdomens bzw. CT von Thorax/Abdomen mit Kontrastmittel Ggf. Hals-Nasen-Ohren-ärztliche Begutachtung (v. a. bei Nikotin- und Alkoholanamnese)
Infektiologisches Screening	Überprüfung des Impfstatus, Ausschluss bzw. Überprüfung der Immunitätslage in Bezug auf bakterielle/virale/fungale/ggf. parasitäre Erkrankungen
Anästhesiologische Beurteilung	Erfassung des Narkoserisikos
Ernährungsmedizinische Konsultation	Erfassung des Ernährungszustands, ggf. Etablierung von Zusatznahrung
Osteoporose-Screening	Densitometrie
Zahnärztliche Abklärung	Ausschluss beherdeter Zähne bzw. enoraler Entzündungsherde
Psychologisch-psychiatrische Evaluation	Erfassung von Suchterkrankungen und psychiatrischen Erkrankungen, Überprüfung von Compliance und Adhärenz

Generell sollte eine Abklärung zur Lebertransplantation immer dann erwogen werden, sofern eine leberzirrhose-assoziierte Komplikation wie Aszites, eine Varizenblutung oder hepatische Enzephalopathie, aufgetreten ist oder eine hepatische Funktionsverschlechterung auftritt, die mittels MELD-Score ≥15 reflektiert wird. Die Abklärung zur Transplantation sollte vorzugsweise am Transplantationszentrum erfolgen. Patientinnen und Patienten mit einem akuten Leberversagen benötigen einen unmittelbaren Transfer in ein Transplantationszentrum.

## Komplikationen nach Lebertransplantation

**Figure 1:**
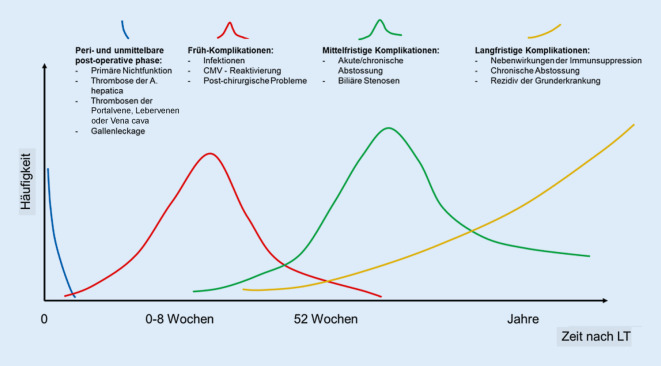

Ein großer Teil relevanter Komplikationen nach Lebertransplantation tritt in der unmittelbaren peri- und postoperativen Phase auf. Hierbei können eine primäre Nichtfunktion des Organs, eine akute Abstossung, schwere Infektionen oder technische Komplikationen, wie eine Stenose und/oder Thrombose der Arteria (A.) hepatica oder Galleleckagen, vorkommen und den Verlauf des Patienten nach Transplantation signifikant beeinflussen. Die häufigsten Komplikationen im Langzeitverlauf betreffen kardiovaskuläre Erkrankungen, de-novo-Malignome oder ein Rezidiv der Grunderkrankung. Einen Überblick über die Komplikationen nach Lebertransplantation gibt Abb. 1.

Die Komplikationen nach Lebertransplantation können wie folgt eingeteilt werden:

- Abstossung, akut oder chronisch
- Technische-operative Komplikationen
- Infektionen
- Folge der langjährigen Immunsuppression
- Rezidiv der Grunderkrankung

## Abstossung

Bei der Transplantation der Leber – genauso wie bei der anderer solider Organe – spielt die akute Abstossung in der postoperativen Behandlung eine zentrale Rolle. Die Prävalenz dieser Komplikation ist sehr variabel (20–30 % der Patienten innerhalb der ersten 6 Monate). Die frühzeitige Diagnose durch eine Leberbiopsie und die umgehende Behandlung einer nachgewiesenen Abstossung sind von großer Bedeutung [4]. Typischerweise zeigt sich eine Erhöhung der Leberwerte v. a. der Transaminasen und γ‑GT ggf. gefolgt von alkalischer Phosphatase und Bilirubin. Die Symptomatik ist normalerweise sehr mild oder fehlend mit allenfalls grippeähnlichen Symptomen, Verschlechterung des Allgemeinzustands und ggf. Ikterus. Selten können sich auch abdominelle Beschwerden entwickeln. Die Diagnose wird durch die Biopsie des Transplantatorgans gesichert. Histologisch zeigen sich drei wesentliche Merkmale: ödematöse und entzündlich infiltrierte Portalfelder, granulozytäre Cholangitis und Endothelialitis. In Abhängigkeit des Schweregrads der akuten Abstossung muss die Immunsuppression erhöht werden bzw. eine Behandlung mit hochdosierten intravenösen Kortikosteroiden (Methylprednisolon) ggf. in Kombination mit Antithymozytenglobulin oder Basiliximab erfolgen.

Die chronische Abstossung weist eine Inzidenz von ca. 2 % pro Jahr auf und spielt vor allem im Langzeitverlauf eine Rolle. Es handelt sich hierbei um eine zumeist T‑Zell-vermittelte, selten antikörpervermittelte Schädigung des Transplantats, die eine weitgehende Zerstörung der Gallengänge sowie der Venen und Arterien zur Folge hat (histologisch: „*vanishing bile duct syndrome*“ und schaumzellige Arteriopathie). Klinisch äussert sich die chronische Abstossung im Spätstadium vor allem mit zunehmender Cholestase, Ikterus und entsprechenden Laborwerten. Die Therapie einer frühen chronischen Abstossung liegt in der Steigerung der Immunsuppression. In fortgeschrittenen Fällen, v. a. wenn bereits ein Vanishing-bile-duct-Syndrom vorliegt, ist leider häufig eine Retransplantation erforderlich.

## Technische-operative Komplikationen

Zu den operationstechnisch bedingten Komplikationen zählen vor allem biliäre und vaskuläre Komplikationen.

Die Prävalenz biliärer Komplikationen in der frühen postoperativen Phase, wie Galleleckagen oder Stenosen im Bereich der Gallengangsanastomose nach Lebertransplantation, variiert zwischen 5 und 25 % und erfordert üblicherweise eine endoskopische (endoskopische retrograde Cholangiographie, ERC, mit Implantation einer Endoprothese) oder chirurgische Revision (Hepatikojejunostomie) [5]. Hiervon abzugrenzen sind nichtanastomotische Gallengangsstrikturen, wie progrediente diffuse Strikturen der intrahepatischen Gallenwege („ischemic-type biliary lesions“, ITBL), die eine Retransplantation erforderlich machen können.

Vaskuläre – arterielle oder venöse – Komplikationen nach Transplantation werden mittels Doppler-Sonographie, Schnittbildgebung oder Angiographie diagnostiziert. Frühe Thrombosen oder Stenosen der A. hepatica treten bei 2,5–9 % aller lebertransplantierten Patienten auf und stellen mit 60 % die häufigsten vaskulären Komplikationen nach einer Lebertransplantation dar [6]. Eine Therapie mittels Revaskularisation (Thrombektomie) kann bei umgehender Diagnosestellung versucht werden. Die Thrombose der A. hepatica ist die häufigste der vaskulären Komplikationen und führt nicht selten zum Transplantatversagen. Im Gegensatz dazu führt eine später einsetzende Stenose bzw. ein langsamer chronischer Verschluss der A. hepatica zu einer Destruktion der intrahepatischen Gallengänge und manifestiert sich mit rekurrierenden Cholangitiden, perlschnurartigen Strikturen der Gallenwege sowie intrahepatischen Abszessen (ITBL). Regelmäßige duplexsonographische Untersuchungen zur Kontrolle der Transplantatperfusion sind daher empfohlen.

Thrombosen der Pfortader sowie der Lebervenen oder der V. cava inferior sind seltenere Komplikationen nach Transplantation, die meist durch hyperkoagulatorische Zustände bedingt sind. Revaskularisation und Antikoagulation können versucht werden, eine Retransplantation ist gelegentlich notwendig.

## Infektionen

Abhängig vom Zeitpunkt nach der Transplantation ist mit unterschiedlichen Infektionen zu rechnen. In der frühen Phase nach Transplantation (v. a. in den ersten 4 Wochen bis 3–6 Monate nach Transplantation) ist mit einer hohen Infektinzidenz zu rechnen, vor allem sind chirurgische Infektionen und nosokomiale Infektionen zu beachten. Bei allen Organtransplantationen sowie bei der Lebertransplantation gehören Infektionen durch das Zytomegalievirus (CMV), Herpes simplex-Virus (HSV), Epstein-Barr-Virus (EBV), und Varizella-Zoster-Virus (VZV) sowie die *Pneumocystis-jirovecii*-Pneumonie zu den häufigsten und wichtigsten opportunistischen Infektionen. Es folgen Pilzinfektionen (z. B. Candida-Spezies, Aspergillus fumigatus) und eine ganze Reihe anderer seltenerer Infektionserkrankungen wie Toxoplasmose, Mykobakteriose und Kryptokokkose. Im Langzeitverlauf ab dem 6. Monat kommt es bei transplantierten Patientinnen und Patienten nicht häufiger zu Infektionen als in der Normalbevölkerung mit Atemwegs- und Harnwegsinfekten als den häufigsten Infektionen. Manifeste Infektionen können jedoch bei immunsupprimierten Patientinnen und Patienten kritischer verlaufen [7].

Der CMV-Infektion nach Transplantation ist besondere Beachtung zu schenken. In Abhängigkeit des Immunstatus der Organempfängerin oder des Organempfängers sowie der Organspenderin bzw. des Organspenders können eine antivirale Prophylaxe für 3–6 Monate bzw. regelmässige CMV-PCR-Tests erforderlich sein [8]. Es gilt, eine Reaktivierung des CMV oder eine Primärinfektion mit CMV zu verhindern bzw. diese möglichst frühzeitig zu erkennen, um letztlich potenziell lebensbedrohliche Komplikationen zu vermeiden. Eine Infektion mit CMV kann asymptomatisch sein, als CMV-Syndrom mit Fieber und grippeähnlichen Symptomen verlaufen oder zu unterschiedlichen Organmanifestationen, wie einer Pneumonie, Hepatitis, Kolitis oder seltener Enzephalitis (= „CMV-Disease“) führen. Die CMV-Hepatitis kann zudem eine wichtige Differenzialdiagnose einer Transplantatabstossung sein.

Das „severe acute respiratory syndrome coronavirus 2“ (SARS-CoV‑2), das am Übergang zwischen dem Jahresende 2019 und dem Jahr 2020 die Coronavirus-Disease-2019(COVID-19)-Pandemie auslöste, gehört nun zu den Infektionen, die auch bei Patientinnen und Patienten nach Lebertransplantation zu berücksichtigen sind, dies allerdings v. a. aufgrund der möglichen Komorbiditäten transplantierter Patientinnen und Patienten. Eine Fallserie von lebertransplantierten Patienten aus der Lombardei ergab, dass Begleiterkrankungen das Risiko für schwerere COVID-19-Verläufe stärker erhöhen als die Immunsuppression selbst. Es wird daher derzeit empfohlen, die Immunsuppression bei COVID-19 nicht unkritisch zu reduzieren [9].

## Folgen der langjährigen Immunsuppression

Zur heutzutage eingesetzten Standardimmunsuppression gehören v. a. die sog. Calcineurin-Inhibitoren (CNI: Cyclosporin A, Tacrolimus), die die Basis der immunsuppressiven Therapie darstellen. Es ist aus heutiger Sicht erforderlich, dass die Immunsuppression ein Leben lang eingenommen wird, um Abstossungen zu verhindern. Diese Substanzen haben jedoch auch Nebenwirkungen, die in der Langzeitbetreuung beachtet und gemanagt werden müssen [10].

Eine Hauptnebenwirkung der CNI stellt die Niereninsuffizienz dar. Postoperativ kommt es in bis zu 78 % der Patienten zu einer akuten Niereninsuffizienz, im Langzeitverlauf entwickeln bis zu 85 % der Patienten eine chronische Niereninsuffizienz, mit der ein signifikant erhöhtes Mortalitätsrisiko vergesellschaftet ist [11]. Es gilt daher, die CNI nach dem Prinzip so hoch wie nötig und so tief wie möglich zu dosieren. Gegebenenfalls ist eine Anpassung oder Umstellung der Immunsuppression erforderlich. Zusätzlich sollten weitere Risikofaktoren für eine chronische Niereninsuffizienz, wie arterielle Hypertonie und Diabetes mellitus, optimal eingestellt sein und nephrotoxische Substanzen möglichst vermieden werden.

Weitere relevante Nebenwirkungen sind der nach einer Transplantation auftretende Diabetes mellitus („Post Transplant Diabetes mellitus“, PTDM), der v. a. mit der Gabe von Tacrolimus und Steroiden assoziiert ist. Ein vorbestehender Diabetes kann sich ebenso unter Immunsuppression verschlechtern. Ausserdem zu beachten sind neurotoxische Nebenwirkungen wie Tremor, Kopfschmerzen, zerebrale Krampfanfälle, neurologische Ausfälle bei Leukenzephalopathie, das Syndrom der posterioren reversiblen Enzephalopathie (PRES) und Psychosen. Darüber hinaus können eine Hyperthrichose (Cyclosporin A), Haarausfall (Tacrolimus), arterielle Hypertonie, Hyperlipidämie, Leukopenie, Thrombopenie und Osteoporose auftreten.

Ausserdem besteht bei Einnahme von CNI ein erhöhtes Risiko für das Auftreten von Malignomen, v. a. Plattenepithelkarzinomen und Basalzellkarzinomen der Haut sowie Lymphomen.

Alternativpräparate zur Basisimmunsuppression können sog. Mammalian-target-of-rapamycin(mTOR)-Inhibitoren (Sirolimus, Everolimus) darstellen. Jedoch auch hier kann es zum Auftreten von Hyperlipidämie, Knochenmarksuppression, Wundheilungsstörungen, Muskel- und Gelenksschmerzen, oralen Aphthen sowie in seltenen Fällen zu einer Pneumonitis kommen.

Im Rahmen mehrerer Studien wurde bis dato untersucht, ob die Basisimmunsuppression nach einer Lebertransplantation abgesetzt werden kann, um damit die Malignomrate zu senken und Langzeitnebenwirkungen zu vermeiden. Das Absetzen der Immunsuppression ist lediglich in etwa 20 % der Fälle erfolgreich, in bis zu 76 % kam es zum Auftreten einer akuten Abstossung, in Einzelfällen auch zu einer chronischen Abstossung mit Verlust des Transplantats [12]. Das Absetzen der Immunsuppression kann daher ausserhalb von Studienprotokollen keinesfalls empfohlen werden.

## Wiederauftreten der Grunderkrankung nach Lebertransplantation

Es ist möglich, dass bestimmte Lebererkrankungen nach Transplantation wieder auftreten, sodass engmaschige Kontrollen erforderlich sind, um dies ggf. frühzeitig zu erkennen.

Patientinnen und Patienten, die eine Lebertransplantation beispielsweise aufgrund eines HCC erhalten haben, benötigen v. a. in den ersten Jahren nach der Transplantation engmaschige bildgebende Kontrollen mittels Schichtbildgebung. Die Rezidivrate für das HCC nach Lebertransplantation liegt bei ca. 10–20 % und hat prinzipiell eine schlechte Prognose, da das Tumorstadium bei Diagnosestellung häufig bereits fortgeschritten ist.

Eine zum Zeitpunkt der Lebertransplantation unbehandelte chronische Hepatitis C tritt in nahezu 100 % der Fälle nach Transplantation wieder auf. Da die Hepatitis C bei immunsupprimierten Patientinnen und Patienten deutlich ungünstiger verläuft und eine Rezirrhose oder cholestatische Hepatitis bereits innerhalb von einem Jahr nach Transplantation auftreten kann, ist eine rasche und effektive Therapie erforderlich, um einen Transplantatverlust zu vermeiden. Daher ist eine Therapie mit den bereits erwähnten hocheffektiven DAA generell bereits vor der Transplantation anzustreben. Patienten mit einer Hepatitis-B-assoziierten Leberzirrhose und florider Infektion bzw. mit Hepatitis-B-assoziiertem akutem Leberversagen benötigen jedenfalls eine Prophylaxe mit Hepatitis-B-Hyperimmunglobulin, die bereits intraoperativ gestartet wird. Im Anschluss bzw. zusätzlich ist eine Reaktivierungsprophylaxe mit einem Nukleosid‑/Nukleotidanalogon mit hoher Resistenzbarriere (z. B. Tenofovir, Entecavir) erforderlich.

Bei Patientinnen und Patienten, die aufgrund einer alkoholassoziierten Leberzirrhose transplantiert wurden, kann es in bis zu etwa 25 % der Fälle zu einem Alkoholrezidiv kommen. Ein Einfluss auf das Langzeitüberleben besteht v. a. dann, wenn es zu einem exzessiven Alkoholkonsum kommt.

Jedoch auch nach Transplantation aufgrund einer NAFLD-assoziierten Leberzirrhose kann es zu einem Rezidiv kommen. In einer rezenten Metaanalyse konnte gezeigt werden, dass die 5‑Jahres-Inzidenz der rekurrenten NAFLD bei 82 % liegt und mit einer erhöhten Mortalität einhergehen kann [13]. Eine Kontrolle der metabolischen Risikofaktoren erscheint daher obligat.

Letztlich besteht v. a. auch bei Patientinnen und Patienten, die aufgrund einer PBC, PSC oder AIH transplantiert wurden, das Risiko eines Wiederauftretens der Grunderkrankung in ca. 15–40 %. Dies ist bei der Wahl der Immunsuppression sowie der Kontrollintervalle zu berücksichtigen.

## Take-Home-Message

Patientinnen und Patienten mit möglicher Transplantationsindikation sollten möglichst frühzeitig im Transplantationszentrum vorgestellt werden. Die Hauptindikationen zur Transplantation haben sich in den letzten Jahren v. a. in Richtung Nicht-alkoholische Fettlebererkrankung und hepatozelluläres Karzinom verschoben. Nach der Transplantation stehen in Abhängigkeit vom zeitlichen Abstand zur Transplantation unterschiedliche Dinge im Vordergrund, die zumeist einen interdisziplinären Zugang benötigen. Im Langzeitverlauf steht v. a. das Management von Nebenwirkungen der Immunsuppression sowie kardiovaskulärer und metabolischer Komorbiditäten im Zentrum.

## Abkürzungen

AIH: Autoimmunhepatitis
ALD: „Alcohol-related liver disease“, alkoholische Lebererkrankung
APDL: Adulte polyzystische Degeneration der Leber
BMI: Body-Mass-Index
CCA: Cholangiokarzinom
CHB: Chronische Hepatitis B
CHC: Chronische Hepatitis C
CMV: Zytomegalievirus
CNI: Calcineurin-Inhibitor
COVID-19: Coronavirus Disease-2019
DAA: „Direct antiviral agents“
EBV: Epstein-Barr-Virus
EHE: Hämangioendotheliom
hATTR: Transthyretin-assoziierte familiäre Amyloidpolyneuropathie
HCC: Hepatozelluläres Karzinom
HSV: Herpes simplex-Virus
ITBL: „Ischemic type biliary lesions“
MELD: „Model of endstage liver disease“
mTOR: „Mammalian target of rapamycin“
NAFLD: „Non-alcoholic fatty liver disease“, nichtalkoholische Fettlebererkrankung
NET: Neuroendokriner Tumor
NSE: „Non-standard exception“
PBC: Primär biliäre Cholangitis
PRES: „Posterior reversible encephalopathy syndrome“
PSC: Primär sklerosierende Cholangitis
PTDM: „Post-transplant diabetes mellitus“
SARS-CoV‑2: „Severe acute respiratory syndrome coronavirus 2“
SE: „Standard exception“
SSC: Sekundär sklerosierende Cholangitis
VZV: Varizella Zoster-Virus
